# Supplementary material for: “It would be better for those of us who have the disease not to be ashamed”: Insights from people living with chronic hepatitis B virus infection and healthcare workers providing HBV care in Kilifi, Kenya
Source: PLOS Glob Public Health. 2025 Oct 31;5(10):e0005279. doi: 10.1371/journal.pgph.0005279 (PMC12578158; doi:10.1371/journal.pgph.0005279)
Supplement: S1 File — 18th June 2024. (DOCX) [file pgph.0005279.s001.docx]

**S1 File. Topic guides for focus group discussions with people living with hepatitis B virus infection (HBV). 18^th^ June 2024**

1. **UNDERSTANDING**
   1. What do you understand about HBV now?
      1. Transmission routes?
      2. Treatment
      3. Do you have any thoughts on how transmission could be stopped? E.g. vaccines, testing being more accessible
   2. Has your understanding of HBV changed since your diagnosis?
   3. Has your lifestyle changed since your diagnosis and how?
   4. Do you feel well informed about your diagnosis?
2. **DESCRIBING HBV**
   1. What word do you use for hepatitis B in your local community?
   2. Are there any other words by which it is known?
3. **REACTIONS TO DIAGNOSIS**
   1. Who have you told about your diagnosis? How have they reacted?
   2. Have you felt isolated/stigmatised/discriminated against?
   3. Do you think the community needs to understand more about HBV?
   4. If yes, how could this be done?
4. **RECEIVING CARE**
   1. Is it easy for you to come to the clinic to get your medication for HBV?
   2. If not, what are the barriers for you? E.g. lost work days, expensive
   3. How do you find taking the medication?
   4. Are there any special things you do to remember to take it every day?
   5. What could be done to improve your treatment? Such as longer supplies of medication, clinics in rural areas, separate hepatitis clinics, shorter waiting times.
5. **HEATLHCARE WORKERS PERCEPTIONS**
   1. How do you feel healthcare workers perceive you when they know you have HBV?
   2. Do you feel treated differently?
   3. Do you feel healthcare workers are well informed about HBV?
