# Supplementary material for: “It would be better for those of us who have the disease not to be ashamed”: Insights from people living with chronic hepatitis B virus infection and healthcare workers providing HBV care in Kilifi, Kenya
Source: PLOS Glob Public Health. 2025 Oct 31;5(10):e0005279. doi: 10.1371/journal.pgph.0005279 (PMC12578158; doi:10.1371/journal.pgph.0005279)
Supplement: S2 File — 14th November 2024. (DOCX) [file pgph.0005279.s002.docx]

**S2 File. Topic guides for focus group discussions with healthcare Workers caring for those living with HBV. 14^th^ November 2024**

1. **UNDERSTANDING**
   1. What do you understand about HBV?
      1. Transmission routes
      2. Treatment
      3. Do you have any thoughts on how transmission could be stopped? E.g. vaccines, testing being more accessible
   2. Were you involved in STRIKE-HBV and has your understanding of HBV changed since the study?
2. **REACTIONS TO DIAGNOSIS**
   1. How do patients react to their diagnosis?
      1. What are they worried about?
      2. Have they heard about HBV before, or is it completely new?
3. **PROVIDING CARE FOR HBV**
   1. What care do you provide for HBV here in Kilifi?
   2. Do you know if HBV care is provided elsewhere in the county?
   3. Do you find many people who are diagnosed are lost to follow up?
   4. What are the barriers to people receiving care and how do you think these could be reduced? (e.g. longer supplies of medication, reducing travel time to clinics, expenses)
   5. Do people often see traditional medicine practitioners for advice?
      1. If yes, what sort of advice to they give?
4. **AWARENESS OF HBV**
   1. Do people in the community know about HBV? What do people say about it?
   2. What words have people used to describe HBV?
   3. Do you think HBV is stigmatised?
   4. How do you think community education about HBV could be improved? (e.g. through religious leaders, CHVs, other community organisations).
