# Supplementary material for: “It would be better for those of us who have the disease not to be ashamed”: Insights from people living with chronic hepatitis B virus infection and healthcare workers providing HBV care in Kilifi, Kenya
Source: PLOS Glob Public Health. 2025 Oct 31;5(10):e0005279. doi: 10.1371/journal.pgph.0005279 (PMC12578158; doi:10.1371/journal.pgph.0005279)
Supplement: S1 Table — (DOCX) [file pgph.0005279.s003.docx]

**S1 Table: Changes in HBV care in Kilifi County due to the STRIKE-HBV study.**

| **Category** | **Prior to STRIKE-HBV** | **Interventions undertaken by STRIKE-HBV** | **Situation after STRIKE-HBV** |
| --- | --- | --- | --- |
| **HBV education for staff** | None provided.  Patients managed in the CCC with no staff training. | Hospital education sessions as part of continuing medical education (CME) and to individual departments, particularly the CCC. | Staff have continued HBV education including reaching out with questions.  KCRH is developing hospital specific guidelines for HBV management. |
| **HBV community education** | None provided. | - Health talks in Kiswahili detailing HBV symptoms and transmission 3 x week in KCRH OPD. - Information leaflets provided (16) - Education for PLWHB combined with peer support sessions at the CCC. | Staff able to provide improved education to PLWHB and antenatal women regarding HBV testing. |
| **HBV testing** | No routine HBV testing either free of charge or paid. No advocacy for testing. | Provision of free HBV testing for non-pregnant adults from March 2023 – June 2024. | HBV testing promoted and offered to antenatal women (although currently at an individual cost) |
| **Peer Support** | No peer support for HBV | Nil specific implemented by STRIKE-HBV, but study staff facilitated peer support sessions initiated by the CCC by providing education. | CCC began peer support sessions for PLWHB during STRIKE-HBV and these are continuing following study completion. |

**S1 Table:** Detailing i) Care for people living with HBV (PLWHB) at Kilifi County Referral Hospital (KCRH) prior to the STRIKE-HBV study; ii) Interventions undertaken by the STRIKE-HBV study and iii) Changes in care for PLWHB after STRIKE-HBV completion. Information leaflets were developed in collaboration with the Hepatitis B Foundation (17). CME sessions mostly targeted doctors, nurses, pharmacists and students. OPD – outpatients department; CCC – comprehensive care clinic.
