# Supplementary material for: “It would be better for those of us who have the disease not to be ashamed”: Insights from people living with chronic hepatitis B virus infection and healthcare workers providing HBV care in Kilifi, Kenya
Source: PLOS Glob Public Health. 2025 Oct 31;5(10):e0005279. doi: 10.1371/journal.pgph.0005279 (PMC12578158; doi:10.1371/journal.pgph.0005279)
Supplement: Attachment — Submitted filename: 250623_Response to reviewers.docx [file pgph.0005279.s006.docx]

Journal Requirements:

1. Please include a complete copy of PLOS’ questionnaire on inclusivity in global research in your revised manuscript. Our policy for research in this area aims to improve transparency in the reporting of research performed outside of researchers’ own country or community. The policy applies to researchers who have travelled to a different country to conduct research, research with Indigenous populations or their lands, and research on cultural artefacts. The questionnaire can also be requested at the journal’s discretion for any other submissions, even if these conditions are not met. Please find more information on the policy and a link to download a blank copy of the questionnaire here: <https://journals.plos.org/globalpublichealth/s/best-practices-in-research-reporting>. Please upload a completed version of your questionnaire as Supporting Information when you resubmit your manuscript.

2. Please send a completed 'Competing Interests' statement, including any COIs declared by your co-authors. If you have no competing interests to declare, please state "The authors have declared that no competing interests exist". Otherwise please declare all competing interests beginning with the statement "I have read the journal's policy and the authors of this manuscript have the following competing interests:"

3. In the online submission form, you indicated that Full recordings and transcripts in both English and Kiswahili of these FGDs are held on a secure server at KWTRP but could be accessed by other studies on request to dgc@kemri-wellcome.org and approval by the data governance committee. For the purpose of Open Access, the author has applied a CC-BY public copyright license to any author accepted manuscript version arising from this submission. This manuscript was written with the permission of Director KEMRI CGMRC.

All PLOS journals now require all data underlying the findings described in their manuscript to be freely available to other researchers, either
1. In a public repository,
2. Within the manuscript itself, or
3. Uploaded as supplementary information.

This policy applies to all data except where public deposition would breach compliance with the protocol approved by your research ethics board. If your data cannot be made publicly available for ethical or legal reasons (e.g., public availability would compromise patient privacy), please explain your reasons by return email and your exemption request will be escalated to the editor for approval. Your exemption request will be handled independently and will not hold up the peer review process, but will need to be resolved should your manuscript be accepted for publication. One of the Editorial team will then be in touch if there are any issues.


[Note: HTML markup is below. Please do not edit.]

Reviewers' comments:

Reviewer's Responses to Questions

**Comments to the Author**

1. Does this manuscript meet PLOS Global Public Health’s [publication criteria](https://track.editorialmanager.com/CL0/https:%2F%2Fjournals.plos.org%2Fglobalpublichealth%2Fs%2Fcriteria-for-publication/1/010f019776dc34fb-80ee83dc-f06c-4cef-8aee-d9745019b9c9-000000/DjYDEPjA5OdpvMQO8OvbrcJNttgEmZ5Tq2E77TsLLhc=215)? Is the manuscript technically sound, and do the data support the conclusions? The manuscript must describe methodologically and ethically rigorous research with conclusions that are appropriately drawn based on the data presented.

Reviewer #1: Yes

Reviewer #2: Yes

2. Has the statistical analysis been performed appropriately and rigorously?

Reviewer #1: N/A

Reviewer #2: N/A

3. Have the authors made all data underlying the findings in their manuscript fully available (please refer to the Data Availability Statement at the start of the manuscript PDF file)?

The [PLOS Data policy](https://track.editorialmanager.com/CL0/https:%2F%2Fjournals.plos.org%2Fglobalpublichealth%2Fs%2Fdata-availability/1/010f019776dc34fb-80ee83dc-f06c-4cef-8aee-d9745019b9c9-000000/IXBiJIyTg3VkEqSgUb78iXLiaONp-2_eLr68KXYAHNs=215) requires authors to make all data underlying the findings described in their manuscript fully available without restriction, with rare exception. The data should be provided as part of the manuscript or its supporting information, or deposited to a public repository. For example, in addition to summary statistics, the data points behind means, medians and variance measures should be available. If there are restrictions on publicly sharing data—e.g. participant privacy or use of data from a third party—those must be specified.

Reviewer #1: Yes

Reviewer #2: Yes

4. Is the manuscript presented in an intelligible fashion and written in standard English?

PLOS Global Public Health does not copyedit accepted manuscripts, so the language in submitted articles must be clear, correct, and unambiguous. Any typographical or grammatical errors should be corrected at revision, so please note any specific errors here.

Reviewer #1: Yes

Reviewer #2: Yes

5. Review Comments to the Author

Please use the space provided to explain your answers to the questions above. You may also include additional comments for the author, including concerns about dual publication, research ethics, or publication ethics. (Please upload your review as an attachment if it exceeds 20,000 characters)

Reviewer #1: This is a well-written study that explores the understanding of HBV infection at both individual and community levels, it examines how the lives of PLWHB have changed since their diagnosis and investigates barriers to care while considering strategies to mitigate them. The study’s objectives are clearly defined, and it effectively delivers on its aims.

Many thanks for your very supportive response to this manuscript, and the value it adds to the voices of those living with HBV and healthcare workers. We are grateful to you for taking the time to review this piece.

However, I have two queries:

1. How did you ensure that responses from focus group discussions were not influenced by the interviewer’s expectations? As noted in the study’s limitations, both healthcare workers and PLWHB have participated in previous research and engaged in peer support groups. How do you account for the potential influence of prior discussions and engagements on their responses? In this regard, would it be beneficial to include the interview questions as supplementary material and reference them in the methods section. This could help demonstrate how the questions encouraged participants to elaborate on their own experiences.

We appreciate the reviewer’s insightful observation. We have added some new sentences to the methods to explain how we minimised the effect of interviewer bias and how we tried to ensure the influence of previous experience in peer support groups and the research study was limited (lines 120 - 130).

We have added a note in the limitations section (lines 527 – 530)

We have also added the topic guide as supplementary material.

2. Regarding participant recruitment, the study mentions that participants were purposively sampled to ensure diversity in age and experience. However, it is unclear whether data collection continued until saturation was reached and whether the selected groups effectively contributed to achieving this. Could this be clarified or acknowledged as a limitation?

We were not able to undertake these discussions until saturation due to time and money constraints within this study. This has been added as a limitation (lines 531-532).

Reviewer #2: Review comments

General observation: This manuscript offers a valuable contribution to the literature on hepatitis B virus (HBV) care in the WHO-AFRO region, particularly by highlighting the perspectives of both people living with HBV (PLWHB) and healthcare workers (HCWs). The study effectively combines qualitative insights to provide understanding of the challenges faced by PLWHB in accessing care, as well as the barriers encountered by HCWs in delivering adequate treatment. The authors offer actionable recommendations for improving HBV care, particularly through community education, decentralization of services, and addressing stigma. The inclusion of both PLWHB and HCWs’ voices enriches the study and makes it a significant addition to the ongoing discussions around HBV management in low-resource settings. Overall, this is a well-executed study that provides practical suggestions for improving care pathways and offers important insights for policymakers and health systems in the region.

Many thanks for your very supportive comments. We really appreciate you taking the time to review this piece in detail and you have suggested some very beneficial changes that we have implemented that hopefully make this piece more robust and of more benefit to the hepatitis B community globally and particularly in WHO- Africa.

The following comments can help improve the manuscript.

Introduction:

• Can the flow between the Kenya-specific situation and broader African context be made smoother for better readability? The paragraph transitions are abrupt. The shift from Kenya’s situation (lines 61–67) to WHO-AFRO and stigma (lines 69–77) feels disjointed. We have rephrased this to make it flow better - lines 67 - 72:

*“As these efforts evolve, it is critical to incorporate both the voices of people living with HBV (PLWHB) and the perspectives of healthcare workers (HCWs), who face operational and systemic challenges in delivering care. These themes are echoed across the broader WHO-AFRO region, where emerging reports highlight the psychosocial burden of an HBV diagnosis and the pervasive stigma experienced by PLWHB.”*

• Briefly elaborate on the HBV stigma survey’s findings or relevance, instead of mentioning it vaguely. We have elaborated on these findings (lines 73 - 75) as below:

*“The World Hepatitis Alliance has piloted a regional HBV stigma survey to better understand these challenges (6). Key findings include PLWHB reporting being avoided at work and in public spaces, feeling they are treated unfairly in healthcare settings and being worried about disclosing their status to families.”*

• The author may consider moving the methodological details (platform building, SRQR compliance) to the Methods section for cleaner structure. The methods are leaking into the introduction.

Thanks for picking up on this - we have moved this into the methods section (lines 92-93).

Methods
Context
• Could you provide more information on why there is no routine laboratory or imaging assessment for HBV treatment eligibility at KCRH? This is important for understanding the limitations in the clinical setting, otherwise readers may wonder why such assessments are not available or why this is the standard practice at the hospital.

We have added a sentence to explain this as below (lines 110 - 112):

*“At KCRH, further laboratory or imaging assessment to determine HBV treatment eligibility is either not available (such as HBV viral load and elastography measurement) or beyond the financial means of most PLWHB (such as complete blood count or liver function tests to calculate APRI score), therefore standard care is that all those testing HBsAg positive receive nucleoside analogue (NA) therapy using tenofovir/lamivudine (TDF/3TC) combination therapy.”*

• The description of HBV care at KCRH could benefit from more detail. It would be useful to explicitly state whether the Comprehensive Care Clinic (CCC) only handles HIV and HBV cases or if it serves other infectious diseases as well. This would provide a clearer understanding of how HBV care fits within the broader healthcare framework at KCRH. The CCC only provides HIV and HBV care - we have added this detail in (lines 102-103)

Data collection
• The dates (e.g., "18th June 2024") should be consistently formatted. Consider either writing the month fully ("18 June 2024") or using the same format throughout. We have ensured this is consistent throughout.

• Could the selection of participants during peer support sessions lead to any potential bias in the representativeness of PLWHB, and how might this affect the findings?

Thank you for raising this important point. We acknowledge that recruiting participants from peer support sessions may introduce a degree of selection bias, as these individuals may be more engaged with care or more open to discussing their experiences compared to the wider population of people living with HBV (PLWHB). This may limit the representativeness of the views captured, particularly in relation to individuals who are less connected to care or support networks.

However, we also found that participants from peer support sessions offered rich insights into the lived experience of HBV, including stigma, disclosure, and navigating the healthcare system—topics that may be difficult to access in more general populations. To mitigate bias, we ensured diversity in age, gender, and healthcare worker cadre and triangulated findings with data from healthcare workers and other sources to support broader interpretation.

We have added a note on this in the limitations section to enhance transparency (lines 527-531)

Data analysis
• You mention that LD developed the coding framework and refined it through discussion with the team. It would strengthen your credibility to briefly explain how disagreements or differences in interpretation were handled.
• Was coding refined iteratively as new data came in (i.e., were earlier codes updated after later FGDs)? If yes, briefly stating this would highlight reflexivity and responsiveness to the data.

Thanks - We have added a new sentence to reflect the iterative nature of data collection and more detail on how the coding framework was developed and refined as below (lines 177 - 179):

*“...the coding framework was first developed by LD and iteratively updated as new data came in. Sections with unclear themes were examined collaboratively with the research team, and differences in interpretation were resolved through group consensus to ensure consistency and strengthen the rigour of the analysis.”*

Results
Knowledge and understanding of HBV
• Paragraphs from lines 168 to 214 are dense. Breaking them into clear sub-sections or signalling sub-themes (e.g., understanding of HBV transmission, perceptions and naming of HBV in the community, Beliefs in bewitchment and alternative treatments) would help readability. Thanks - we have included some sub-headings into this section to improve readability.

• Some redundancy in quotes: A couple of quotes echo the same point (e.g., HBV perceived as fatal/unknown). One or two could be trimmed to avoid repetition and improve narrative flow.

We have trimmed down some of the quotes to reduce repetition, whilst trying to ensure there is as wide a representation of views as possible.

Comparison with HIV

• The second theme "Comparison with HIV" is accurate but broad. The author may consider a more descriptive title like: "Confusion with HIV, stigma spillover, and funding disparities between HBV and HIV" or anything deemed better.

Thanks - we have changed the title of this section to ‘confusion with HIV and stigma spillover’ (line 238) make it more explanatory.

How lives have changed since HBV diagnosis

• "How lives have changed since HBV diagnosis" is accurate but can be more analytic. Something like: “post-diagnosis transformations: emotional distress, lifestyle change, and altered life plans." We have changed this as suggested (line 257-258).

• The paragraph/section is currently long and hard to navigate. Breaking it into subthemes would greatly enhance clarity. Thanks - we have broken this up into different headings to improve navigation through the paragraph.

Barriers
Economic factors

• The last two quotes (on stigma and difficulty asking for fare) feel somewhat tacked on. A transition sentence summarizing the tension between proximity and stigma would smooth the flow.

We do feel we have already written a transition sentence to show the tension between proximity and stigma as below: *“Several people in both the patient and HCW groups mentioned that (despite the potential higher cost) some PLWHB would prefer to go to a distant clinic (where they are not known) to reduce stigma. However sometimes asking for money for transport caused problems with questions about where they were going”*

Stigma and lack of education

• The first quote feels abrupt due to missing transition or setup. A lead-in sentence clarifying the context of self-stigma at home would improve flow. We have removed the first few words of the quote to make it more readable (lines 397-398). It now reads: *“I can't eat with them at the same table, it is as if I will infect them, I should have my own bowl and sit aside”, (participant 3, PLWHB, female, FGD 1).*

Reducing transmission

• The idea that education is key is repeated several times. A concise synthesis paragraph summarizing these points once, rather than reiterating throughout, might improve readability.

We have summarised the findings around improving education to gather all these suggestions into one place just before the discussion (lines 437-467).

Discussion
• Tables are labelled at the top - We have inserted an up to date version of this table which is hopefully easier to interpret.

• Ensure consistency in how references are cited—e.g., use either "(23,24)" or "[23,24]" uniformly, based on your chosen style guide. We have ensured this is consistent.

• Some sentences are long and slightly convoluted. Example:
"Several suggestions from participants here on how to improve care are already included in national Kenyan guidelines…"
Could be revised to: "Several participant suggestions align with national Kenyan HBV management guidelines, such as antenatal HBV testing and vaccinating close family members…"

Many thanks - we have been through the discussion and re-written a few longer sentences to be more concise.

• Minor grammatical error on line 486

Limitations
• Fix typo in the abbreviation on line 504

Note: I have incorporated some comments in the PDF version of the manuscript.

Thank you and all the best.

6. PLOS authors have the option to publish the peer review history of their article ([what does this mean?](https://track.editorialmanager.com/CL0/https:%2F%2Fjournals.plos.org%2Fglobalpublichealth%2Fs%2Feditorial-and-peer-review-process%23loc-peer-review-history/1/010f019776dc34fb-80ee83dc-f06c-4cef-8aee-d9745019b9c9-000000/pG4rv6rAFOqKMkZ7yo3E5OI_PCbl451PwZyVpwLP7x8=215)). If published, this will include your full peer review and any attached files.


**Do you want your identity to be public for this peer review?** If you choose “no”, your identity will remain anonymous but your review may still be made public.


For information about this choice, including consent withdrawal, please see our [Privacy Policy](https://track.editorialmanager.com/CL0/https:%2F%2Fwww.plos.org%2Fprivacy-policy/1/010f019776dc34fb-80ee83dc-f06c-4cef-8aee-d9745019b9c9-000000/GYq1gjAEkdSE8EQJeKA9WUZP0LobKKE5pRWkJtwCoiU=215).

Reviewer #1: No

Reviewer #2: No
