# Supplementary material for: “It would be better for those of us who have the disease not to be ashamed”: Insights from people living with chronic hepatitis B virus infection and healthcare workers providing HBV care in Kilifi, Kenya
Source: PLOS Glob Public Health. 2025 Oct 31;5(10):e0005279. doi: 10.1371/journal.pgph.0005279 (PMC12578158; doi:10.1371/journal.pgph.0005279)
Supplement: Attachment — Submitted filename: 250916_Response_to_reviewers.docx [file pgph.0005279.s007.docx]

6. Review Comments to the Author

Please use the space provided to explain your answers to the questions above. You may also include additional comments for the author, including concerns about dual publication, research ethics, or publication ethics. (Please upload your review as an attachment if it exceeds 20,000 characters)

Reviewer #2: The authors have adequately addressed all my comments from the previous review, providing clear revisions and justifications where necessary.

Thank you.

Reviewer #3: The authors report the findings of a qualitative study detailing the experiences of PLWHB and HCWs who care for PLWHB from a single hospital in Kenya. Overall, the results are interesting and informative but this study is limited by its small sample size. Specific comments for improvement are available below.

Specific Comments

Overall:
- The writing style has many “run-on” sentences that could be shortened for brevity and clarity
- The current manuscript has well over 6,000 words. This needs to be reduced substantially to less than 4,000 words.

Many thanks for your comments. We have reduced the ‘run on’ sentences where possible and condensed the whole manuscript down as much as we can. We are keen to keep the quotes from those who took part in the study as this is the best representation of how HBV affects those living with the virus and how healthcare workers manage HBV. There is so little information in this space globally, we think it adds an important aspect to the manuscript.

Introduction

- Sentence starting with “HBV has, however…” reads awkwardly. Consider something like “Despite this global burden, HBV has been neglected…”

This been altered and now reads: “HBV has been neglected in terms of healthcare provision, education, research and policy (2,3) and it is estimated only 4.2% of those living with HBV in WHO-AFRO have been actually diagnosed.”

- Sentence starting with “an estimated 4.2%...” is unclear. Do you mean that 4.2% of all people have HBV or that only 4.2% of all estimated patients with HBV have been diagnosed?

This has been rephrased and now reads “HBV has been neglected in terms of healthcare provision, education, research and policy (2,3) and it is estimated only 4.2% of those living with HBV in WHO-AFRO have been actually diagnosed.”

- Line 64: you state “little vaccination outside of the childhood immunisation series”. Most children who have received their childhood immunization series do not need additional doses. Do you mean that HBV vaccines are unavailable to adults who did not receive their childhood HBV series?

This has been rephrased and now reads: “There is minimal population screening and vaccine is mostly unavailable to adults at risk of exposure (such as healthcare workers) who were not vaccinated as children.”

- Sentence line 70-71: please provide citation – done

- Sentence line 75-77: you already say something similar in lines 66-68.

We have reduced sentence 75-77 to just make the broader point of how those with lived experience can help improve advocacy, a separate point to lines 66-68.

- Condense sentences from 77-82 into a single sentence

We have condensed this section to be more concise and it now reads:

“The positive role of peer support workers in HBV care is being increasingly recognised, to encourage testing, improve treatment adherence and reduce stigma (10). Healthcare workers (HCWs) providing care for PLWHB have a unique insight into barriers to long-term follow-up and medication. They understand the healthcare infrastructure for PLWHB, have insights into gaps and challenges, and can advise how access could be improved.”

- Sentence 84-86: This sentence needs to be revised. I would start with “We aimed to describe the experiences of PLWHB and HCWs caring for PLWHB in Kilifi County Referral Hospital (KCRH) in Kenya, to help inform care delivery. We also sought to establish a scalable platform for qualitative data collection that would be useful in other settings.”

This has been altered as suggested.

Materials and Methods
- I’m not sure you need a whole table 1 to describe the facilities in Kilifi County. This information does not seem directly relevant to your paper.

We agree. This has been removed.

- Table 2 does not directly relate to what was conducted in this study. This could be presented in a supplement, but should not appear in the main body of the manuscript.
This has been moved to supplementary material.

Results
- Table 3 should be explicitly referred to in the results section

We have now referred to this table at the beginning of the results section to give a summary of the findings.

- Consider cutting back significantly on the number of words in this section

We have reduced the number of words in this section where possible, however we would like to keep the quotes as they are to give the most accurate representation of the opinions of both people living with HBV and healthcare workers.

Discussion
- Line 620-622: Earlier in the manuscript you state that not all participants preferred decentralization, because it could actually worsen stigma. So I think this sentence has to be edited to reflect that it should be decentralized for those who wish to have services closer to home.

We agree with this and have altered the sentence to now read:

“However, we suggest that caution is needed and whilst decentralised clinics should be available to those who want services closer to home, extensive community education and sensitisation should be done before this is implemented and services further afield should be maintained.”
